# When the Nail Gun Goes Wrong: A Case of Penetrating Globe Injury

**DOI:** 10.7759/cureus.79894

**Published:** 2025-03-01

**Authors:** Taylor Cesarz, Michael Thompson, Lance Barnett, Shayne Gue

**Affiliations:** 1 Department of Emergency Medicine, University of Wisconsin School of Medicine and Public Health, Madison, USA; 2 Department of Emergency Medicine, University of Central Florida/HCA Florida Healthcare (Greater Orlando/Osceola), Kissimmee, USA; 3 Department of Emergency Medicine, BayCare Health System/St. Joseph's Hospital, Tampa, USA; 4 Department of Medical Education, University of Central Florida College of Medicine, Orlando, USA

**Keywords:** emergency medicine, globe rupture, open globe, penetrating ocular injury, seidel sign, vitreal penetration

## Abstract

Globe rupture is a rare but sight-threatening ocular emergency that requires prompt recognition and management by emergency medicine providers. We report the case of a 27-year-old male who presented to the emergency department following accidental ocular trauma from a nail gun, resulting in classic physical exam findings consistent with globe rupture. Examination revealed a positive Seidel sign, confirming the presence of an open-globe injury. The patient underwent management in the emergency department, including measures to prevent elevated intraocular pressure, administration of tetanus prophylaxis, initiation of broad-spectrum antibiotics with vitreal penetration, and urgent ophthalmology consultation. Globe rupture is a time-sensitive diagnosis requiring a high index of suspicion and rapid intervention to preserve visual function. Emergency medicine practitioners must be adept at recognizing key clinical findings, implementing critical initial management steps, and coordinating immediate ophthalmologic evaluation to optimize patient outcomes.

## Introduction

Ocular injury is a relatively common cause of emergency department visits, prompting 2.5 million visits annually in the US, with a higher frequency of injuries occurring in late spring (April through June) [1,2]. However, open-globe injuries account for a much smaller percentage with an estimated incidence of three per 100,000 [3]. Of these, an overwhelming majority of patients are male, comprising roughly 80% of cases and most often occurring between ages 10-30 years old [4,5]. Women have a much lower incidence of open globe injuries, occurring most frequently in elderly patients who have recently undergone ocular procedures such as cataract removal or corneal transplants [6,7].

The mechanism of injury can vary widely, but patterns have been identified amongst certain patient groups. Pediatric cases often involve penetrating trauma from pencils/pens, vegetation/sticks, air-powered rifles (pellets, paintball guns), and fish hooks. Additionally, blunt trauma usually involves contact with sports equipment (balls, pucks, or other players) [8]. In young adult males, blunt trauma is usually from assault with fists or other blunt objects [5]. Other adult cases are often associated with workplace injuries from construction sites, factories, farms, and carpentry. These are usually penetrating injuries from projectile material such as concrete, stone, wood, nails, equipment, and other debris. The use of safety glasses dramatically reduces this risk, as most patients report not wearing eye protection at the time of injury [5,9]. Elderly women may present after a fall, resulting in blunt trauma to a previous ocular surgical site [6,7]. Motor vehicle collisions (MVCs) may also cause penetrating or blunt trauma in all patient populations, especially incidents involving broken windows, deployed airbags, and unrestrained passengers [10]. The use of alcohol or other intoxicating substances is a risk factor in most cases, especially those involving MVC, assault, and workplace injuries [9,11].

Injuries involving laceration, puncture, or rupture of the globe are very serious and may result in permanent vision loss or life-threatening infection if not promptly diagnosed and treated. Often, these injuries are treated directly in the emergency department. Blunt trauma usually results from abrupt compression of the eye within the bony orbit. Orbital fractures may be involved. Injury to the eye commonly occurs proximal to the insertion of the rectus muscle, insertion of the optic nerve, limbus, or prior surgical sites [12].

Penetrating trauma involves the cornea in two-thirds of cases and results from foreign objects puncturing or lacerating the eye [13]. Intraocular foreign bodies may be present and increase the risk of infection, especially those involving organic matter or resulting in delayed primary closure beyond 24 hours. In such cases, infection rates can be as high as 13% [14].

## Case presentation

Patient information

A 27-year-old man, with no major past medical history, was brought in by emergency medical services as a transfer from a local low-resource community emergency department for left eye pain in the setting of ocular trauma. The patient reported that he accidentally shot himself in the eye with a nail gun six hours prior to arrival while he was examining it close to his face, as it had jammed. After firing the nail gun into his eye, he pulled the nail out himself. He reported pain and complete loss of vision in the affected eye. He denied falling, head strike, or additional trauma. There was no history of contact lens use or glasses.

A 12-point review of systems was negative other than what was described in the history of the present illness. He denied all past medical and surgical history. He denied any medication use. He was initially seen at a community emergency department without access to specialized ophthalmologic care and was transferred to our facility for further evaluation of ocular trauma.

Clinical findings

The patient's vital signs on arrival were as follows: heart rate of 84 beats/minute, respiratory rate of 19 breaths/minute, oxygen saturation of 100% on room air, blood pressure of 123/67 mmHg, and temperature of 36.9°C.

**Figure 1:**
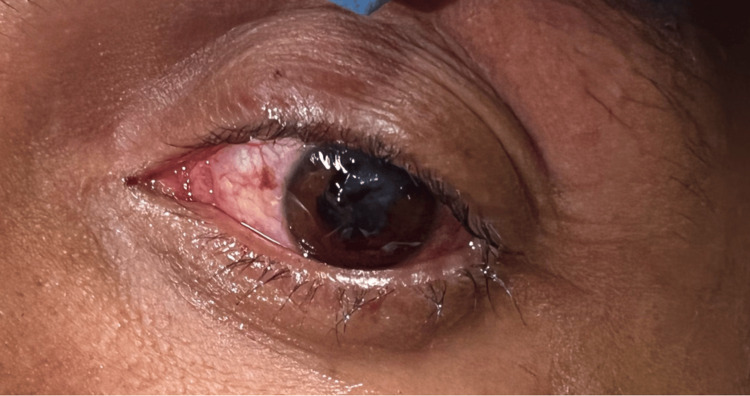
Conjunctival injection and pupillary deformity

Physical exam findings included an awake, alert patient with an obvious deformity and non-reactivity of the left pupil and left conjunctival injection (Figure 1). His visual acuity was reduced to motion and shadows in the affected eye. Extraocular movements were intact. The right eye was without injection and had 20/20 visual acuity. No other facial deformities or signs of head trauma were noted on the exam.

Diagnostic assessment and therapeutic testing

Laboratory evaluation performed at the outlying facility revealed a leukocytosis of 14,800 cells per microliter (reference range: 4,500-11,000 cells per microliter). His hemoglobin level, electrolytes, creatinine level, and liver function tests were within normal limits. He received intravenous piperacillin-tazobactam, vancomycin, morphine, and intramuscular tetanus vaccination.

**Figure 2:**
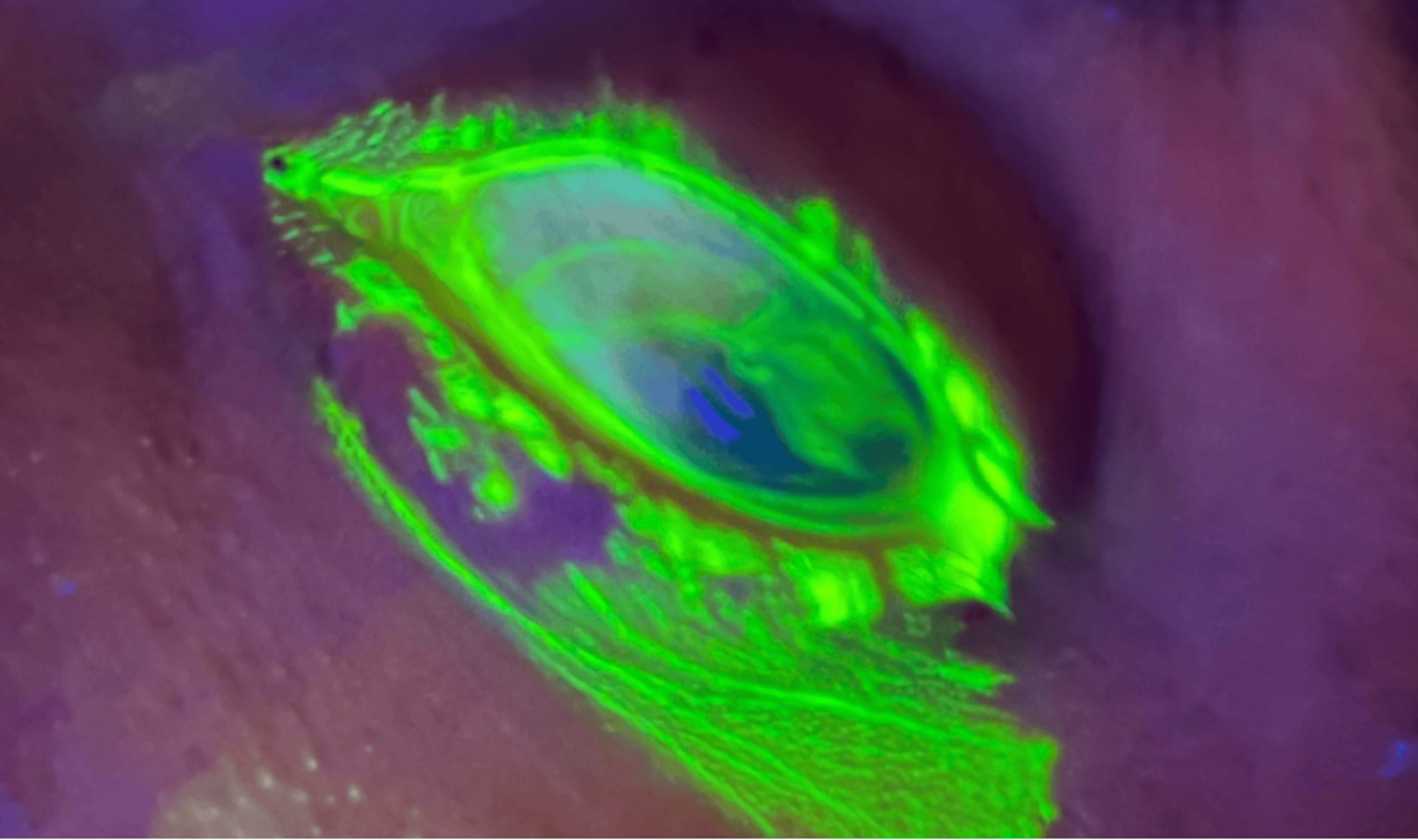
Fluorescein stain with Wood’s lamp exam showing extrusion of fluid consistent with a positive Seidel sign

On arrival at our emergency department, we performed a fluorescein stain and Wood’s lamp exam with the results pictured in Figure 2.

**Figure 3:**
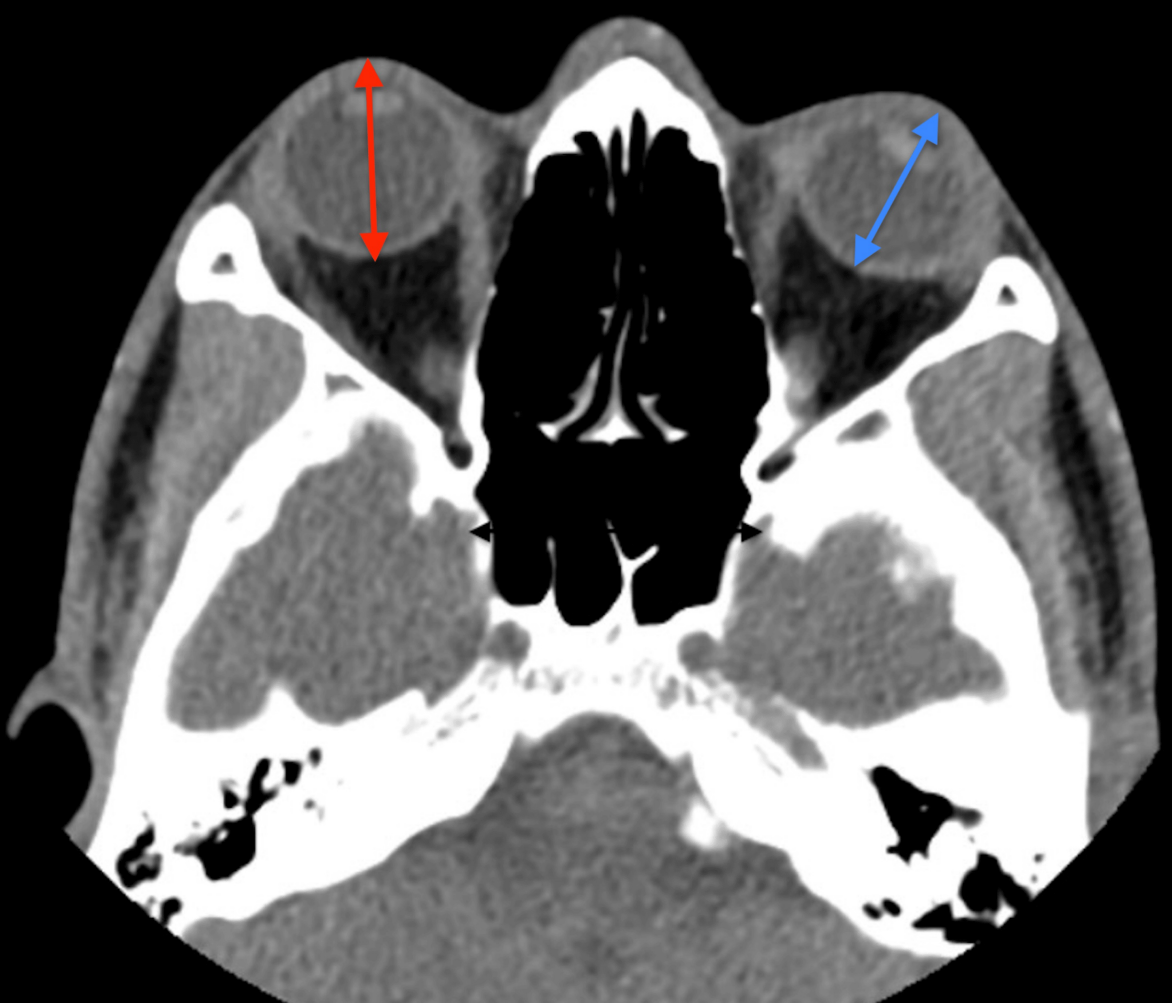
CT orbit without contrast revealing normal anteroposterior diameter of the right orbit (red arrow) and shortening of the anteroposterior diameter of the left orbit (blue arrow), confirming globe rupture

We administered intravenous ceftazidime, applied a fox shield, elevated the head of the bed, and consulted ophthalmology. Ophthalmology recommended a computed tomography (CT) scan of the orbit without contrast which was significant for shortening of the left globe without any evidence of retained foreign body (Figure 3). The patient was admitted inpatient to the trauma service for surgical evaluation.

Follow-up and outcomes

The patient was taken to the operating room on the day of admission for globe repair and uveal tissue repositioning. He was discharged on postoperative day one. He was discharged with prednisolone acetate 1% eye drops, timolol maleate 0.25% eye drops, and neomycin/polymyxin B/gramicidin 1.75 mg-10,000 units-0.025 mg/mL eye drops. He was recommended to follow up with ophthalmology on an outpatient basis two days after discharge.

The patient returned to our emergency department 18 days after hospital discharge for left eye pain for a few days duration. The patient had difficult access to outpatient follow-up care with ophthalmology related to logistical healthcare difficulties. He was noted to have conjunctival injection, visual acuity to shadows (the same postoperatively), normal intraocular pressure measurement of 18 mmHg, and no evidence of erythema, discharge, bleeding, or pus. The ophthalmologist who performed the repair was consulted and recommended that no further acute intervention would be required in the hospital. The patient was subsequently discharged.

## Discussion

Globe rupture is an ophthalmologic emergency that emergency medicine providers must know how to recognize and manage. Diagnosis of globe rupture should include a focused history evaluating the mechanism of injury, any other associated traumatic injury, and tetanus status [3]. In addition to performing an initial trauma survey, a detailed ocular exam should be performed. Visual acuity testing is important to assess vision deficits. Visual field deficit testing should be performed [15]. A pupillary exam should be performed, assessing for any pupillary deformity and an afferent pupillary defect [3]. Findings of a teardrop-shaped pupillary deformity should raise suspicion for globe rupture [15]. A slit lamp exam can reveal corneal lacerations, penetrating foreign bodies, or prolapse of ocular structures [3]. The fluorescein staining test is of particular use in the emergency department because it is relatively quick and easy to perform. The eye should be stained with fluorescein dye, followed by the use of either a slit lamp or Wood’s lamp, to examine for streaming aqueous humor indicative of corneal laceration [15,16]. This test, while not sensitive, if positive, is specific for globe rupture [3].

The role of imaging in the diagnosis of globe rupture is adjunctive. While point-of-care ultrasound has much utility in diagnosing many different eye-related emergencies, it is contraindicated in cases of suspected globe rupture, as direct pressure may increase the risk of ocular extrusion [3]. CT of the orbits may show findings of globe deformity, intraocular hemorrhage or gas, or changes in anterior chamber depth to help diagnose globe rupture [17]. However, many studies have shown that a CT scan alone is not reliable in diagnosing globe rupture, reinforcing the need for a thorough ocular exam [17,18]. A CT scan is useful for evaluating any remaining penetrating foreign body [3].

Management of globe rupture involves emergent ophthalmology consultation [15]. An eye shield should be placed over the eye, either a fox shield or a paper cup [15]. Care should be taken to prevent the shield from putting pressure on the eye. Manipulation of the eye should be avoided [15]. Avoid any processes that involve direct ocular pressure, including eyelid inversion, tonometry, and ocular ultrasound [3]. Maneuvers that reduce intraocular pressure should be employed, including elevation of the head of the bed, antiemetics to avoid increased intraocular pressure due to vomiting, and pain control [15].

One of the major complications of globe rupture is post-traumatic endophthalmitis, and tetanus vaccination should be updated to reduce this risk [18]. Prophylactic antibiotics should be given, ensuring that antibiotics with intravitreal coverage are chosen [19]. Common organisms causing endophthalmitis after globe rupture include *Staphylococcus*, *Bacillus*, *Streptococcus*, *Pseudomonas*, and *Clostridium* species [19,20]. Common antibiotic regimens include ceftazidime and vancomycin. Intravitreal antibiotics should be considered in high-risk cases, including retained intraocular foreign body and soil contamination [19]. While antibiotics play an important role in preventing this serious complication of globe rupture, the most important intervention is emergent primary globe repair, making emergent ophthalmologic consultation a cornerstone of globe rupture management [19,20].

## Conclusions

It is essential for emergency medicine providers to be able to recognize and manage globe ruptures. Emergency medicine physicians should be aware of physical exam maneuvers to clinically diagnose globe rupture and should have knowledge of how to properly manage these serious injuries, including measures to avoid elevation of intraocular pressure and emergent ophthalmology involvement in care.
